# Assessing ecosystem service trade-offs and synergies in the rapidly urbanizing coastal region of Mangaluru Agglomeration, India

**DOI:** 10.1371/journal.pone.0344106

**Published:** 2026-03-23

**Authors:** Deeksha Nayak, Anoop Kumar Shukla

**Affiliations:** Manipal School of Architecture and Planning, Manipal Academy of Higher Education, Manipal, Karnataka, India; CEPT University, INDIA

## Abstract

The steady growth of the cities alters the urban environmental pattern and functions, posing signs of danger mainly on the ecosystem services. Mangaluru is a coastal city, where rapid urbanization is seen presenting both opportunities and challenges for sustaining the vital ecosystem services. In this study, we utilized InVEST (Integrated Valuation of Ecosystem Services and Trade-offs) model, a widely spread, open-source tool employed to map and quantify the benefits of ecosystem services. Water yield, carbon sequestration and soil retention are the services assessed in this study. Furthermore, we applied Ecosystem Service Trade-offs and Synergies Degree Index (ESTD) to evaluate their relationships and analyzed how various policies affected land cover and ecosystem services of Mangaluru during 1980–2022. The results showed that built up area increased at the expense of wasteland/shrubland, and agricultural land. Notable increase in water yield is observed. Carbon storage and soil loss has reduced in the study area. Between these ecosystem services, water yield presented a clear trade-off relationship with soil loss, while carbon storage was synergistic with soil loss. Our findings show that different policies have diverse consequences on the environment and ecosystem services. Under economic policies, built-up areas developed dramatically, resulting in the loss of natural land and carbon storage. Ecological policies increased water yield, and a large amount of wasteland/shrubland land was transformed into forest. The study’s conclusions will help policymakers balance economic development and environmental conservation.

## 1 Introduction

The burden on ecosystem services (ES) is increasing because of socioeconomic and climatic variables. According to researchers [1,2], climate change may have a detrimental effect on both service provisioning and regulation as the world’s population increases. For human well-being, natural ecosystems provide critical functions like carbon sequestration, climate management, biodiversity conservation, and aesthetic delight [3–5]. Furthermore, due of the trend of global warming, climate change has caused a number of ecological problems, such as resource scarcity and frequent severe weather [6,7]. Trade-offs and synergies are the two types of connections that exist between ecosystem services, which are dynamic. Synergies occur when there are positive correlations between ES; trade-offs occur when there are negative correlations. When taking into account a number of factors, such as biology, climate, and the complex character of ecosystems, researchers found that there is frequently seasonal and regional fluctuation in the interactions between ES [8–10].

LULC has been demonstrated to be among the primary determinants of ES changes [11]. The extent of LULC is significantly impacted by human activity, particularly in the case of various land cover-governed regulations being put into place [12–14]. On the other hand, ecosystem deterioration [15], biodiversity loss [16], and changes in water yield [17] were all inevitable outcomes of urbanization and industrialization.

Scholars who conduct significant studies on LULC and hydrology-related topics have always attempted to comprehend the relationship between different ESs, mostly concentrating on sediment yield, soil loss from erosion, water yield, and the impact of climate change in conjunction with shifts in LULC [18]. Changes in temperature and precipitation, which have significant implications on water availability and quality, have an adverse influence on ecosystems and the services they offer [19,20].

Modelling of the ES helps the researcher to quantify, spatially locate, and potentially evaluate the economic trends. Integrated Valuation of Environmental Services and Trade-offs (InVEST), is a globally accepted tool which was developed inside the Natural Capital Project [21–24]. InVEST model can illustrate a spatially visualized map of the ESs. InVEST model is being used extensively due to its input data criteria, which requires low amounts of data and low levels of expertise, thus making it acceptable worldwide; it uses open source data that are freely available, with a mapping/modelling scale of 30 m × 30 m. This model helps us to access multiple ecosystem services, (water quality, soil erosion, carbon sequestration, biodiversity conservation, nutrients, agricultural produce, etc.) this model provides an accurate assessment with limited demand of data input criteria’s and is relevant in understanding the areas dealing with ecological processes [23,25,26]. InVEST model is a useful tool to assess small-scale and local studies which gives relevant and credible results for LULC and ES [21]. To capture the intensity of changes taking place at the regional level, the study is in line with the SDG 2030 given out by UN Habitat, along with the spatio-temporal parameter addressed.

In this study, we analysed the influence of guidelines on the ES of Mangaluru from 1980–2022. Specifically, we (1) applied the InVEST model to measure the subtleties of various ES and used ESTD to gauge their relations; (2) analysed the influences of economic and ecological rules implemented on LULC and ES. The results are supportive for promoting sustainable enhancement and decision makings in Mangaluru.

## 2 Study area

**Fig 1:**
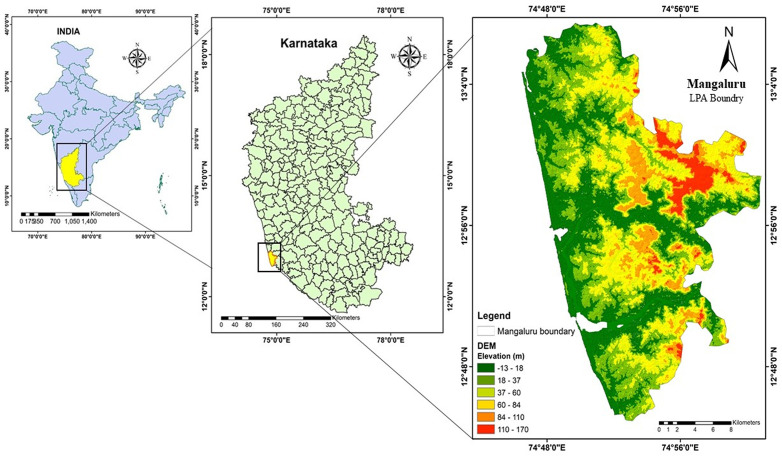
Location of study area (a) India boundary (b) Karnataka State Boundary and (c) Mangaluru Agglomeration boundary. (Created using ArcGIS software (version 10.8); data are freely available at https://earthexplorer.usgs.gov/).

The study area is situated on the western coastal belt of India (Fig 1). The study area is defined by the natural determinants like, Arabian sea on the west side, ecological hotspot, i.e., Western ghats on the east side. It is one of the major port towns situated on the western coast, housing more than half a million people, i.e., 623841, due to which the study area falls under category of class I towns under census 2011. We find two major water bodies on the northern and southern sides of the central business district or the urban core of the study area, namely Gurupura and Netravathi. Mangaluru Taluk is a Municipal Corporation unit of Dakshina Kannada district, which houses two Municipal Councils, and 1 Town Panchayat.

The extent of Mangaluru taluk (study area) is around 567 sq.km, extending from 12º 48’ 0’‘ N to 13º 4’ 0’‘ N and 74º 48’ 0’‘ E. Annual precipitation of 3,695 mm, with maximum and minimum temperature of 34.5 ºC and 16 ºC is recorded in the study area. Built up areas are rapidly growing because of urbanization in Mangaluru. There is research which highlight on the unstable and unsustainable type of urban growth taking place in the study area, which emphasis on work required to comprehend the intricacy of urbanization, in order to enable urban designers, interpret intricate urban sceneries and accomplish sustainable urban expansion [20,27,28].

Mangaluru, a place that has been proposed as one of the future IT hubs, it is imperative to investigate the corresponding growth of its social economy and environmental conservation. Previous studies on the Mangaluru mostly examined changes in ES or how land cover affected ES [20,27,28]. Few attempts have been made to look closely at the ES and LULC policies. However, it is currently unclear how various policies affect LULC and ES, which makes it challenging to support future advancements and changes in this field. Therefore, Mangaluru desperately requires a detailed examination of how various policies affect LULC and ES.

## 3 Ecosystem services quantification

Considering the data available in our research area, we defined three ecosystem services: water yield (WY), sediment loss (SL), and carbon storage (CS). Considering the amount of precipitation in the study area we found it necessary to assess the WY factor. Since the study area is proposed to a new IT hub, it is important to understand the CS and SL in the study area to protect the environment. Rapid urbanization has significantly changed the terrain into an urban area since 1980, which has had an immense effect on WY and CS. Three ES were quantified in the current research using the InVEST model. A popular tool for assessing and studying ES is the InVEST (Integrated Valuation of Ecosystem Services and Trade-offs) model, which can quantify different ES and display them as a graph [29,30].

### 3.1 Water yield

The InVEST Water Yield model calculates the proportional contributions of water from various regions of a landscape, revealing how modifications to land use patterns impact yearly surface water yield and hydropower output.

Budyko curve (1974) is employed for water yield model, using annual precipitation. The model computes the total yearly water yield (Y) for every pixel (x) in the study area using the following formula:


$$\text{Y}(\text{x})=\left(\text{1}-\frac{\text{AET}(\text{x})}{\text{P}(\text{x})}\right)\cdot \text{P}(\text{x})$$ (1)

Where, AET (x) = annual actual evapotranspiration,

P(x) = annual precipitation on pixel x.

The model considers that, on a yearly time scale, every drop of water pouring as precipitation above a catchment region, subtracting the evapotranspiration, departs the watershed. There is no differentiation made among surface and subsurface water movement.

In practice, estimating the region of interest and determining the scale of annual real evapotranspiration is quite challenging. Evaluating evapotranspiration at the landscape scale is tough due to the need for advanced equipment and methodologies at the plot and field scales [12,31,32].

The evapotranspiration portion of the water balance, $\frac{AET(x)}{P(x)}$ for vegetated land use is calculated

Zhang et al. [33] in a spatially explicit way on pixel x by


$$\frac{\text{AET}(\text{x})}{\text{P}(\text{x})}=\text{1}+\frac{\text{PET}(\text{x})}{\text{P}(\text{x})}-{\left[\text{1}+{\left(\frac{\text{PET}(\text{x})}{\text{P}(\text{x})}\right)}^{\omega }\right]}^{\frac{\text{1}}{\omega }}$$ (2)

Where, PET (x) = annual potential evapotranspiration

ω(x) = parameter characterizing climate-soil properties.

The formula for obtaining PET (x) and ω(x) are as follows:


$$\text{PET}(\text{x})={\text{K}}_{\text{C}}\left({\text{l}}_{\text{x}}\right)\times \text{E}{\text{T}}_{\text{0}}(\text{x})$$ (3)

$$\omega (\text{x})=\text{Z}\frac{\text{AWC}(\text{x})}{\text{P}(\text{x})}+\text{1}.\text{25}$$ (4)

where, AWC(x) = plant available water content

Z = Zhang coefficient (depends on number of rain events per year),

ET_o_ = reference evapotranspiration

K_c_(l_x_) = evaporation factor for each LULC.

For other land use types such as water bodies, build-up lands AET(x) is calculated directly from reference evapotranspiration (ET_o_) as


$$AET(x)=\left(K_{C}\left(l_{x}\right)\times {ET}_{o}(x).P(x)\right)$$ (5)

#### 3.1.1 Average annual reference evapotranspiration.

The Hargreaves equation (1985) was used to calculate the annual reference Evapotranspiration. The average yearly reference evapotranspiration in raster format produced by the IDW interpolation.

#### 3.1.2 Plant available water content.

The percentage of water that may be kept in the soil profile and made available to plants is known as the plant available water content, or PAWC. PAWC was computed in ArcGIS from AWC raster data acquired from International Soil Reference and Information Center (ISRIC) SoilGrids.

#### 3.1.3 Soil depth.

Average soil depth in raster format was created using Soil Survey of India records with the help of GIS software. At 1–5 m soil depth, we find traces of laterite, red sandy loam and alluvial soil.

#### 3.1.4 Land use land cover.

The LULC maps were generated using ArcGIS 10.2.2 from satellite data acquired from United states geological survey (USGS) earth explorer portal. The method involved object-based image analysis (OBIA) classification with Nearest Neighbor (NN) technique. The LULC was classified in five distinct classes, namely, Agricultural land, Built up area, Forest, Water bodies, Shrubland/wasteland. The Landsat imagery for 5 decades, 1980, 1990, 2000, 2010, 2022 was used to generate the LULC.

#### 3.1.5 Precipitation and watershed.

The gridded data from Indian Meteorological Department (IMD), Pune, yearly data of (0.25x0.25) degree, for the temporal period of 1980, 1990, 2000, 2010, 2022. The IDW interpolation method in ArcGIS was used to create the annual mean rainfall raster value in mm. The study area received an average of 3,695 millimeters (145.5 inches) of rainfall between 1980 and 2022. The months of June through September see the most precipitation, with July having the highest average rainfall of 1,059 millimeters (41.7 inches). ArcGIS was used to create watersheds based on the digital elevation model (DEM). The ws_id is a unique identification number assigned to each watershed.

#### 3.1.6 Biophysical table.

LULC classes with information on biophysical coefficients are labeled by a biophysical table. These criteria are established with reference to FAO, ISRIC, ICAR-NBSSLUP, and IPCC, and the data represents features of each LULC class.

#### 3.1.7 Seasonality factor (*Z*).

According to Donohue et al. [34], Z is a Zhang coefficient [35], an empirical constant that represents the hydrogeological features and local precipitation pattern. According to Hamel and Guswa [36], it was calculated as 0.2*N, where N is the average number of rainy days (>1 mm) that occurred during the research period. Consequently, 22 is determined to be the Z value of the study area.

### 3.2 Carbon storage

In this study, we estimated the total carbon storage of the region using InVEST carbon sequestration model. The LULC map and the four carbon concentrations that relate to the various LULC were used by the model to determine the outcomes. Soil carbon, dead organic carbon, and above- and below-ground carbon are some of these carbon concentrations. Consequently, the following formula is used to determine the total carbon storage:


$$C_{total}=C_{above}+C_{below}+C_{soil}+C_{dead}$$ (6)

### 3.3 Soil loss estimation

A spatially explicit model operating at the input DEM raster’s spatial resolution is the InVEST sediment delivery ratio (SDR) module [37]. To assess the formation of overland sediment and its transport to streams or rivers, we used the SDR model, considering data accessibility and model uncertainty [37].

#### 3.3.1 Soil loss estimation.

The SDR model initially uses the revised universal soil loss equation (RUSLE) to calculate the yearly soil erosion for a specific pixel at the pixel level. The SDR factor, which is described as the percentage of soil loss that reaches streams, was then multiplied by the yearly soil loss, which is impacted by factors including climate, land management, terrain, and soil composition. The following calculation was employed to estimate the mean annual SL rate:


SL = R * K * LS * C * P (7)

Where, SL (t ha^-1^year^-1^) = annual soil loss,

*R* (MJmm (ha h year) ^−1^) = rainfall erosivity,

*K* (t ha h (MJ ha mm) ^−1^) = soil erodibility,

LS (unitless) = topographic factor,

*C* (unitless) = cover management factor,

and *P* (unitless) = supporting practice factor.

The computation was done at the pixel level, and the quantity of each pixel’s value is used to determine the soil retention service [38].

### 3.4 Ecosystem service trade-offs and synergies degree index (ESTD)

To comprehend ES, we’ve employed ESTD to describe the relationship that connects them. ESTD can characterize the nature and number of interactions between several ESs. As a result, it is widely used to study the relationship between several ES [39,40]. The ESTD index is established as:


$$ESTD_{ij}=\frac{ESC_{ib}-ESC_{ia}}{ESC_{jb}-ESC_{ja}}$$ (8)

𝐸𝑆𝑇D_𝑖𝑗_ = trade-off or synergy degree between *i* and *j*. If ESTD is larger than 0, it signifies synergistic, else trade-off; 𝐸𝑆𝐶_𝑖𝑎_ and 𝐸𝑆𝐶_𝑖__𝑏_ indicate the values of the 𝑖 ES at time 𝑎 and 𝑏, respectively 𝐸𝑆𝐶_𝑗𝑎_ and 𝐸𝑆𝐶_𝑗__𝑏_ represent the values of the 𝑗 ES at period 𝑎 and b, respectively. To reduce magnitude difference, we normalized the 𝐸𝑆𝐶 values from 0 to 1, reflecting the lowest and maximum levels of ES, respectively.

## 4 Result

### 4.1 LULC changes

The classification may be seen on the map and covers water bodies, forest, agricultural land, wasteland/shrubland, and built-up regions in the decadal study. Forests are the main factor influencing LULC categorization, followed by built-up area and agricultural land, according to the decadal analysis. Out of all the cover classes, the water body makes up the smallest percentage.

**Fig 2:**
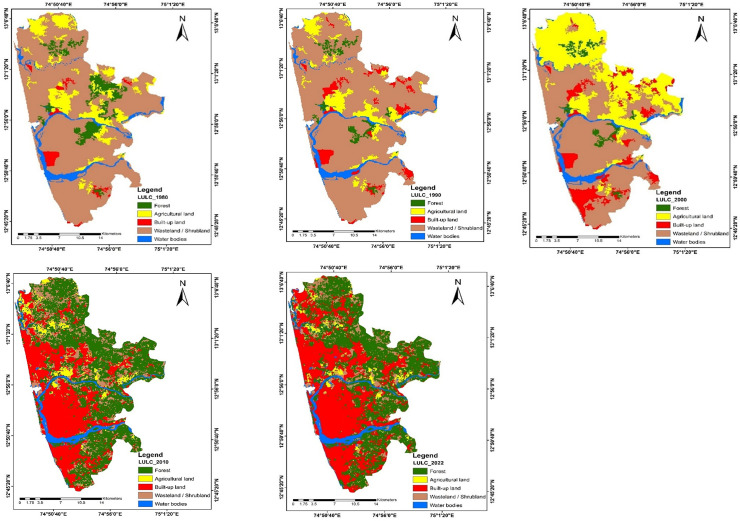
LULC maps of Mangaluru in 1980, 1990, 2000, 2010, and 2022 (Created using ArcGIS software (version 10.8); data are freely available at https://earthexplorer.usgs.gov/).

To precisely define LULC, multi-temporal images collected via satellite were employed. Between 1980, 1990, 2000, 2010, and 2022, the investigation illustrates the spatial changes in the LULC classes; agricultural land, forest area, water body, built-up area, and barren land (Fig 2). There is a significant variation in Agricultural land which decreased by 9.54%. In contrast, there was a notable gain in built-up area of 37.10%, a decrease of 63.44% in wasteland, a 35.83% increase in forest areas, and a 0.06% increase in water bodies (Table 1). Due to spatial determinants, it is worth to note that the spatial expansion in the region is taking place in North- South direction and on the East direction. The land-use transition pattern provides a comprehensive understanding of how the landscape within the Mangaluru Agglomeration has transformed over the 42-year period from 1980 to 2022. While Table 2 summarizes changes in total area under each LULC category, the transition pattern reveals how these changes occurred by quantifying conversions from one class to another. This pixel-based interpretation is essential for understanding the drivers of landscape change and the consequences for ecosystem services, urban expansion, and environmental sustainability. A key observation from the pattern is the significant conversion of wasteland/shrubland areas into built-up land. In 1980, wasteland/shrubland covered approximately 41,478 ha, but by 2022, only 5,379 ha remained. The transition pattern suggests that a substantial portion of former wasteland—over 24,000 ha—was transformed into built-up areas, making this the dominant land conversion pathway in the region. This reflects the rapid urbanization that has taken place over the last four decades, driven by population growth, industrial expansion, and infrastructure development. The transformation of wasteland/shrubland into residential, commercial, and industrial land uses highlights the increasing demand for land in the urban core and peri-urban zones. Another notable transition is the marked decline in agricultural land, which decreased from 7,184 ha in 1980 to only 1,756 ha in 2022. The pattern indicates that a considerable portion of agricultural land—over 6,000 ha—transitioned into built-up land. This indicates a steady encroachment of urban infrastructure onto cropland, driven by economic restructuring and changes in livelihood patterns. The marginal areas of agriculture also shifted into shrubland or fallow land during intermediate decades before being converted to urban use, reflecting a transitional phase often observed in rapidly urbanizing regions. The dynamics of forest cover show a more complex pattern. Forests decreased sharply between 1980 and 2000 but increased substantially by 2010 and remained high in 2022. The pattern shows conversions of forest to built-up land and wasteland during earlier years, but also significant gains through afforestation or reforestation initiatives, particularly after 2000. Approximately 12,000 ha of land transitioned into forest by 2022, largely from former wasteland areas, the reason being the ecological plantation drive for the costal belt of Karnataka “Karavali Hasiru Kavacha Yojane” in the year 2000, along with Costal Biodiversity Conservation and Mangrove restoration drive which played major role in increasing the green cover of the study area. However, the transition pattern also reveals that the new forest cover is more likely to consist of young or plantation forests, which has implications for carbon storage and ecosystem service provisioning. Water bodies, on the other hand, remained relatively stable over the study period, with only minor fluctuations caused by local hydrological modifications or small-scale encroachments. The transition pattern shows minimal conversions from water bodies to other land uses or vice versa, indicating that water features remained largely preserved despite intense urban pressure. Overall, the land-use transition pattern offers deeper insights into the spatial patterns and processes of landscape transformation. It reveals that urban expansion has been the dominant driver of change, primarily consuming agricultural and shrubland areas. Forest gains result mostly from land reclamation or restoration rather than conservation of existing forests. These transitions have far-reaching implications for ecosystem services, including water yield, carbon storage, soil retention, and urban ecological resilience. Understanding these pathways helps to contextualize the ecosystem service assessments and highlights the need for sustainable land-use planning in the Mangaluru Agglomeration.

**Table 1:** Spatial Coverage of LULC classes in Mangaluru city.

LULC Class	1980		1990		2000		2010		2022	
	Area (ha)	(%)	Area (ha)	(%)	Area (ha)	(%)	Area (ha)	(%)	Area (ha)	(%)
Water bodies	2949	5.18	2949	5.18	2978	5.23	2890	5.08	2985	5.25
Forest	3923	6.89	3712	6.52	1767	3.11	25162	44.22	24309	42.72
Wasteland/Shrubland	41478	72.90	40233	70.71	27837	48.92	7724	13.58	5379	9.45
Agricultural land	7184	12.63	6980	12.27	18772	32.99	2631	4.62	1756	3.09
Built-up land	1364	2.38	3019	5.31	5541	9.74	18490	32.50	22470	39.49
**Total (ha)**	56898	100	56898	100	56898	100	56898	100	56898	100

**Table 2:** LULC Transition Pattern (1980–2022) in hectares.

1980 → 2022	Water Bodies	Forest	Wasteland/ Shrubland	Agricultural Land	Built-up land	Total 1980
**Water bodies**	2600	50	80	40	179	2949
**Forest**	90	1500	300	120	1913	3923
**Wasteland/Shrubland**	110	12000	5000	250	24118	41478
**Agricultural land**	50	400	600	90	6044	7184
**Built-up land**	135	359	0	0	870	1364
**Total 2022**	2985	24309	5379	1756	22470	56898

### 4.2 Ecosystem services quantification

#### 4.2.1 Water yield.

**Fig 3:**
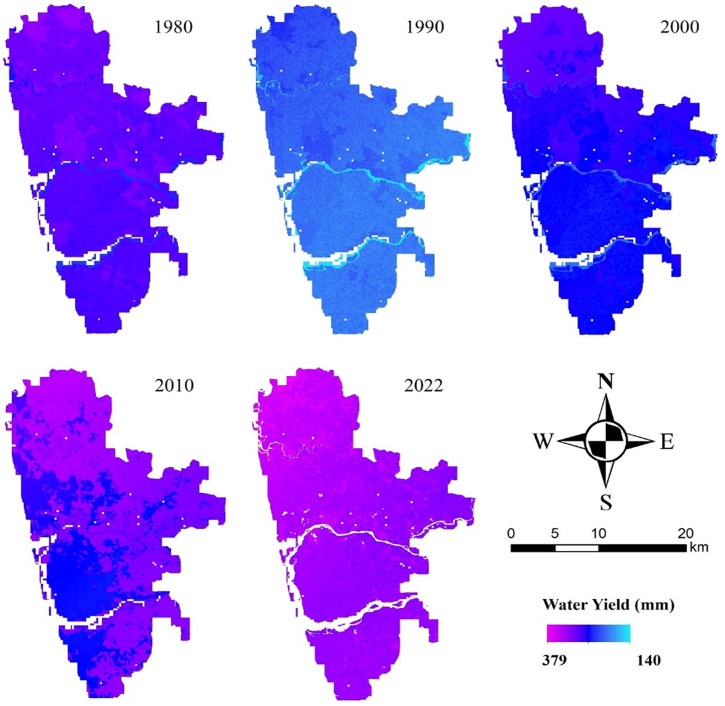
Water yield in 1980, 1990, 2000, 2010, and 2022 (Created using ArcGIS software (version 10.8) with rainfall data from IMD and LULC derived from Landsat images. All datasets are freely available at https://www.imdpune.gov.in/cmpg/Griddata/Rainfall_25_NetCDF.html and https://earthexplorer.usgs.gov/).

The InVEST model is used to obtain the WY pattern of the study area (Fig 3.). The mean water yield in the study area of 1980, 1990, 2000, 2010 and 2022 were 291.10 mm, 208.42 mm, 265.87 mm, 298.94 mm, 328.92 mm respectively. Considering the spatial perspective, areas with low water yield mostly occur on the western coastal belt of the study area, whereas high water yield is distributed throughout the study area, majorly on the northern, eastern side of the study area.

#### 4.2.2 Carbon storage.

**Fig 4:**
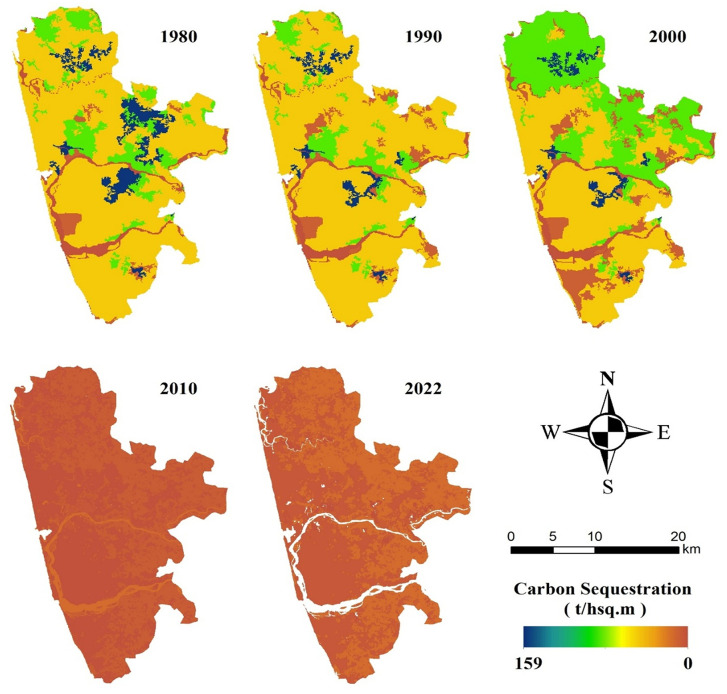
Carbon storage in 1980, 1990, 2000, 2010, and 2022 (Created using ArcGIS software (version 10.8) with LULC derived from Landsat images and Biophysical table. All datasets are freely available at https://earthexplorer.usgs.gov/ and https://soilgrids.org/).

Using InVEST, we obtained the geographical distribution of CS in Mangaluru from 1980 to 2022 (Fig 4.). The regions with the highest carbon storage concentration were found on the eastern boundaries of the study region, as they were largely occupied by agricultural and forest land, both of which have high soil carbon density. In contrast, wasteland/shrubland had slightly lower carbon sequestration capacity, and built-up regions, as expected, had no CS. The region’s CS in 1980, 1990, 2000, 2010, and 2022 were 57.10 Tg C, 50.62 Tg C, 57.29 Tg C, 4.30 Tg C, and 8.94 Tg C, demonstrating a significant drop in overall quantity. Expedited development has facilitated the transformation of wasteland/shrubland to built-up areas, resulting in an alarming reduction in carbon storage from 1980 to 2022 (48.16 Tg C).

#### 4.2.3 Soil loss estimation.

**Fig 5:**
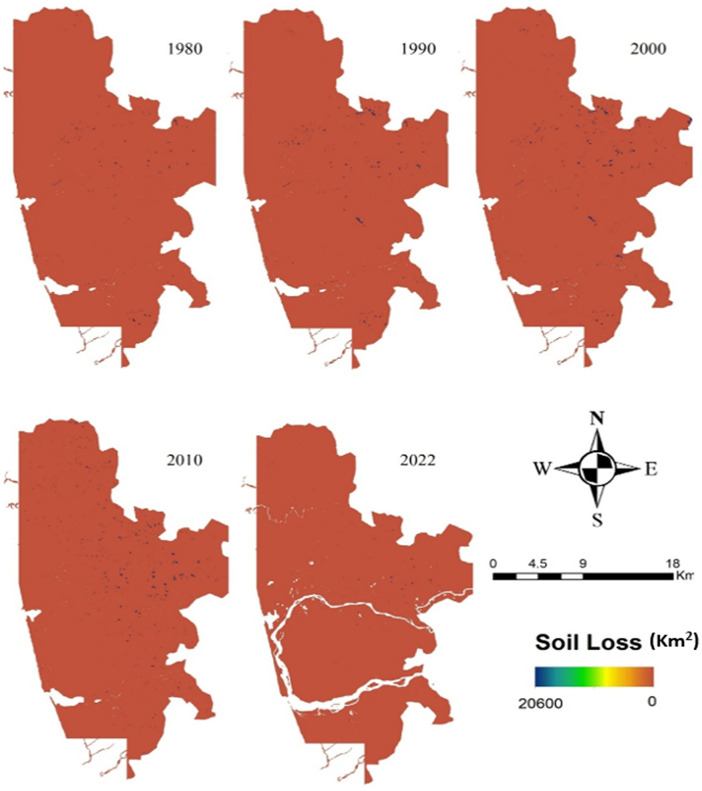
Soil loss in 1980, 1990, 2000, 2010, and 2022 (Created using ArcGIS software (version 10.8) with rainfall data from IMD, LULC derived from Landsat images and Digital Elevation Model (DEM). All datasets are freely available at https://www.imdpune.gov.in/cmpg/Griddata/Rainfall_25_NetCDF.html and https://earthexplorer.usgs.gov/).

An overview of SL classes and their spatiotemporal changes between 1980 and 2022 is shown in (Fig 5). The data reveals that areas experiencing slight SL decreased during the study period. Mean Soil loss during 1980, 1990, 2000, 2010, 2022 is 0.05, 0.05, 0.07, 0.08, 0.04 km^2^ respectively. The mean SL change rate of the research area is very negligible.

### 4.3 Synergy and trade-off analysis between ecosystem services

**Fig 6:**
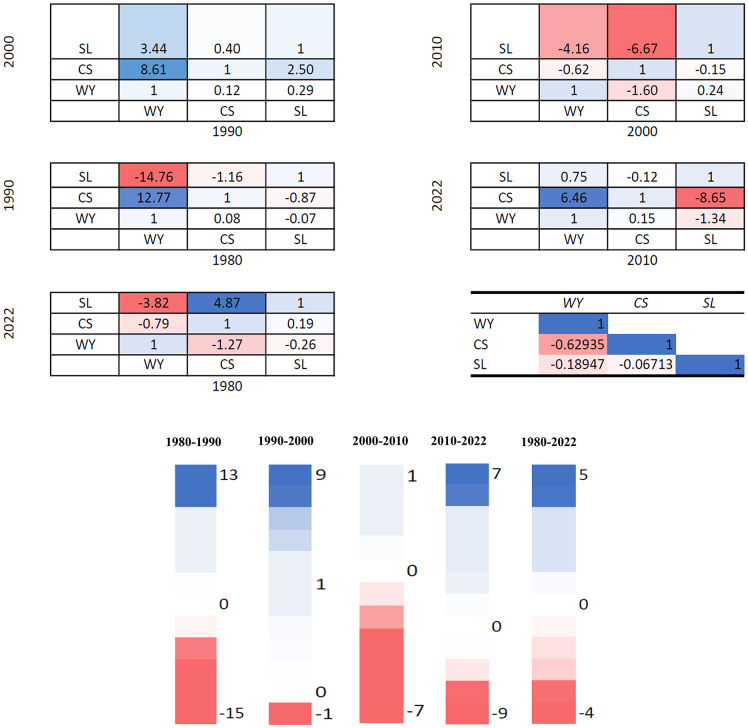
The ESTD between three ecosystem services.

Relationship among the three ecosystem services for four decades is represented in Fig 6, the calculation for the same is based on formula (8). During 1980–1990, the degree of trade-off between WY and SL was 14.76. The same occurred between CS and SL respectively. Between 1990–2000, we find synergy among all the ecosystem services, given that there is decrease in the degree of synergy between WY and CS. Furthermore, during 2000–2010, we find the degree of trade-off between CS and SL was 6.67. The same occurred between WY and SL, CS and WY respectively. Between 2010 and 2022, the synergy between CS and WY further weakened. A trade-off relationship was identified between SL and CS, SL and WY with 8.65 and 1.34. In general, CS and SL had the same trend of change with a degree of synergy of 4.87 and there was a continued trade-off relationship between WY and SL.

## 5 Discussion

### 5.1 Major policy drivers of land cover changes

**Fig 7:**
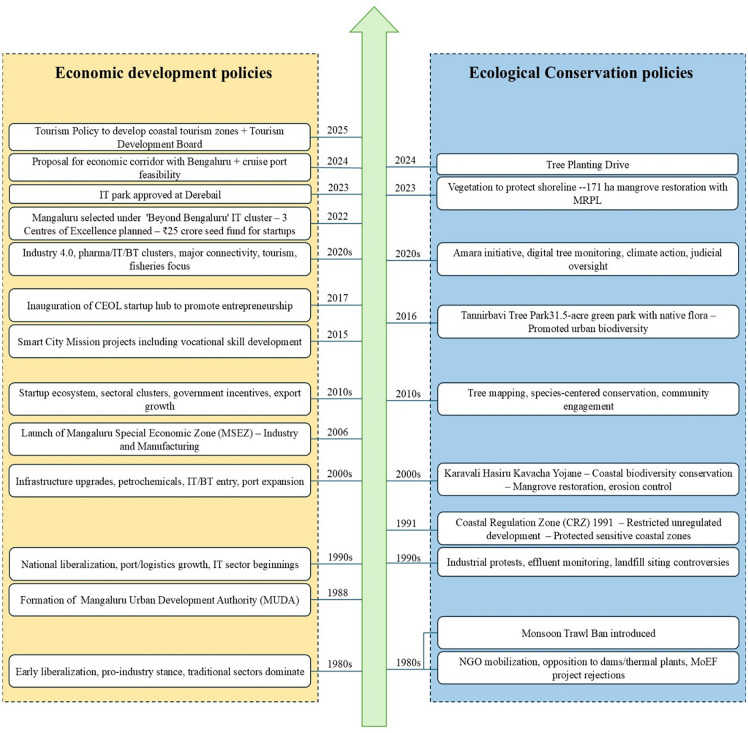
The main economic and conservation policies implemented in Mangaluru from the 1980s (Source: Mangalore Urban Development Authority (MUDDA) website).

Between 1980 and 2022, Mangaluru’s land cover changed dramatically (Table 2). We identified the important national macro policies and regional land policies implemented in Mangaluru over the last forty years and investigated their relationship with land use (Fig 7).

After the 1980s, Mangaluru has implemented a number of economic growth initiatives. The establishment of the urban development body is crucial to the economic development of the city, and it was supported by the construction of IT sectors in the 1990s, which resulted in the expansion of the built-up area south of the study area. Since the establishment of Mangaluru’s special economic zone, the city has been working to become the new Bangalore by hosting IT infrastructure. Rapid urbanisation, fuelled by increasing populations and economic growth, has a profound impact on land cover. Since 2000, as economic development intensified, more workers have continued to relocate to cities, with Mangaluru’s built-up region having the largest growth.

Economic initiatives have aided in growth, but they have also resulted in several environmental concerns. Enforcement of national environmental laws, such as the Forest Conservation Act (1980), the Water (Prevention and Control of Pollution) Act (1974), and the Wildlife Protection Act (1972), are important steps. These laws have established a legal framework for preserving biodiversity, controlling water pollution, and safeguarding forested areas. Innovative measures like nature-based solutions, such as wetland restoration, have been implemented by local government (Fig 7). As we find notice the change pattern from the maps, we find that there is positive outcomes from the government initiatives which improved the green cover of the study area.

To ensure the area’s long-term development, regulations should be updated to reflect patterns in economic growth and environmental resilience. The ecological safety of water must be our first focus. Floods are common owing to the geographic position and the climate’s high rainfall amounts. We argue that the ecology should not be sacrificed for urbanization. Mangaluru is now experiencing problems in its growth. To progress the city-building process objectively, continued research into urban-rural connectivity must enhance the level of urbanization and sustainable development capabilities even further.

The current study examined policy-driven changes in LULC and ES in Mangaluru between 1980 and 2022. However, there are certain limitations to consider. First and foremost, the current study only includes three types of ES, which is insufficient for a full assessment of ES in Mangalore. Second, the model’s findings tend to be ambiguous as a result of limited data availability. In addition, without actual measurement data or variable validation, InVEST parameters like evapotranspiration coefficients and root depths are calculated using empirical formulas or published results. Future research into the mechanisms of ES interactions could benefit from field trials, data sources, and other elements.

## 6 Conclusions

The current study examined policy-driven patterns in land use and ecosystem services in Mangaluru between 1980 and 2022. The region has undergone significant land use shifts. Urbanisation led to the loss of agricultural land and wasteland/shrubland. The built-up area changed significantly, particularly along the city’s North-South-East route. Agricultural land decreased by 9.54% over this time period, according to changes in land use and land cover (LULC). In contrast, forested areas increased by 35.83% as a result of the Karnataka government’s “Karavali Hasiru Yojane” tree planting initiative. Water bodies grew by 0.06%, whereas built-up areas expanded by 37.10%, owing to market value fluctuations between 2000 and 2010, resulting in a 63.44% decrease in wasteland. Overall, the region’s WY and SL increased, whereas CS declined significantly. Using the ESTD data, we discovered that SL produced a clear trade-off with WY whereas CS strengthened their synergy. Our findings demonstrated that urbanisation accelerated the conversion of natural land into built-up regions, resulting in a large loss of carbon storage. Furthermore, climatic conditions have a greater impact on water yield than changes in land cover or constraints. Overall, we anticipate that these findings will improve our understanding of the relationship between policies and ES in Mangaluru, as well as provide important scientific recommendations for future policy development and adjustment.
